# Growth and competitive interaction between seedlings of an invasive Rumex confertus and of co-occurring two native Rumex species in relation to nutrient availability

**DOI:** 10.1038/s41598-019-39947-z

**Published:** 2019-03-01

**Authors:** Jeremi Kołodziejek

**Affiliations:** Department of Geobotany and Plant Ecology, University of Lodz, 12/16 Banacha St., 90-237 Lodz, Poland

## Abstract

*Rumex confertus* is an alien invasive perennial plant that has increased its range rapidly within central Europe in the last 100 years. This study examined the effects of a commercial fertilizer on the competition between the invasive *Rumex confertus* and two non-invasive native species *R*. *acetosa* or *R*. *conglomeratus* in terms of morphological and physiological traits and relative yield. All three *Rumex* species were grown in the open field with two levels of nutrient availability in field plots. Competition and fertilizer had significant effects on height, relative growth rate (RGR), specific leaf area (SLA) as well as shoot and root biomass of all three species. The fertilized plants had high macronutrient and nitrate contents in leaf tissue. Relative yield of *R*. *confertus* was <1, indicating that for this species the effects of interspecific competition were greater than those of intraspecific competition. The results of this experiment indicate that there is interaction between the nutrient status of the soil and the competition between species. Competitive superiority of *R*. *confertus* could explain its dominance in grasslands and in disturbed areas, and might explain its great influence on the occurrence of native species because competition intensity was high in fertilized plots.

## Introduction

Competition is likely to be an important determinant of plant community structure and is considered one of the most important factors promoting successful invasive potential^1,2^. Some invasive species are competitive in relation to native species, with the strongest competition occurring between species with similar ecological niches and/or closely related species^3,4^. With respect to invasion, several researchers^5,6^ have suggested that the main factors identified to date contributing to plant invasiveness explaining habitat invisibility are evolutionary history, habitat disturbance, propagule pressure, abiotic stress, soil nutrient availability and community structure. The degree of invasion of habitats was correlated with overall availability of nutrients^6^, and of specific nutrients such as nitrogen (N)^7^, potassium (K)^8^, and phosphorus (P)^9^. The theory of fluctuating resource availability^6^ proposes that the invasibility of a plant community increases as resource availability increases. Due to the fact that invasive species are usually found in disturbed areas such as agricultural fields, where disturbance reduces competition for nutrients or nutrient enrichment may occur, it can be assumed that they will be the most invasive in nutrient-rich habitats. Agricultural nutrient loading in the form of N, P, and K reaches wetlands via grounder water, surface flow, overland flow and precipitation^10–12^. Burke & Grime^13^ manipulated fertilizer in limestone grassland and showed that invasion was strongly related to availability of nutrients. Other studies have showed that hydrological disturbance affected nutrient availability because nitrates were readily leached from oxidized soil during drainage^14^. Anthropogenic nutrient inputs to the biosphere from fertilizers and atmospheric pollutants now exceed natural sources and enhance plant invasion^15^. There is also evidence that invasive species may access forms of nutrients that neighbouring (native) species are not using, including amino^16^.

Nitrogen is limiting nutrient important to fast-growing plants, especially to invasive plants^17^. Experimental studies showed greater relative performance of invasive species than of native ones in N-enriched treatments, implying reduced success of invasives relative to natives in N-poor sites^18–20^. Anthropogenic N enrichment due to of agricultural activities or roadside pollutants was shown to increase the number of N-loving invasive species in relation to native ones in N-poor environments, such as coastal, calcareous and sandy grasslands^21^. Numerous studies on competition demonstrated that excess fertilization and manure production on agricultural lands created surplus N, which was mobile in many soils and often leached to downstream aquatic ecosystems, or volatilized to the atmosphere, could be redeposited elsewhere eventually reaching aquatic ecosystems^11^. Many successful invaders, for example Australian *Acacia* spp., *Myrica faya*, *Leucaena leucocephala*, in N-limited ecosystems, have symbiotic associations with N-fixing bacteria^22^.

Potassium is an important macronutrient in plants needed in a number of crucial metabolic processes including photosynthesis and respiration^8^. A few studies indicated the relationship between invasion success and high K availability in soils^8^. Other studies, however, showed an opposite pattern. The invasive success of *Taraxacum officinalis* in the Andes was linked to low soil K availability^23^.

Eutrophication of most freshwater ecosystems is caused by over enrichment with nutrients, principally phosphorus^9^. The main sources of excess phosphorus inputs to aquatic ecosystems include domestic treated sewage, industrial discharges, storm drainage, and runoff from agriculture^24^. Normally, because phosphorus availability (primarily as orthophosphate) is the limiting element for freshwater macrophytes^25^, reducing phosphorus levels might help in controlling competitiveness of invasive macrophytes^24^.

Comparison of leaf area, plant height, above- and below-ground dry weight have been used to provide information on aggressiveness of species which might influence their competitive ability^26–28^. Some studies found that invasive species had higher biomass than non-invasive ones at high nutrient availabilities and that invasive and non-invasive species did not differ at low nutrient availabilities^29,30^. Competitiveness is often defined by relative yield, i.e. the ratio of yield in mixture to that in monoculture. In mixed swards, the genotype with the higher relative yield is regarded as more competitive^31^.

While many study focused on the relative competitive strength of alien or exotic vs native species^32–34^ and the influence of nutrient availability on invasive success^35,36^, relatively few studies examined experimentally the combined effects of competition and resource availability. Such studies are necessary in order to understand the ability of invasive plants to spread in new areas^37–39^.

Many invasive species are considered competitively superior to co-existing native species, with the strongest competition expected between species with similar niches. In this study a field experiment with pair-wise competition treatments of three *Rumex* species widespread in central Europe, i.e. an invasive alien *R*. *confertus* Willd. and two native species *R*. *conglomeratus* Murr. and *R*. *acetosa* L was performed. It is of interest to understand the ecological interactions among the *Rumex* species because *R*. *confertus* is highly invasive in Central Europe, it has spread on meadows and grasslands along river valleys in eastern Europe. Recently, however, *R*. *confertus* has colonized roadsides and wastelands^40–42^. In the majority of published *R*. *confertus* studies^42,43^, the response to nutrient condition was not investigated.

The competitive ability of a species is a combination of two components: competitive effect, the ability of a species to affect other species, and competitive response, the ability of a species to avoid being affected^44^. In the presented nutrient addition field experiments, a full-factorial randomized complete-block design was used for all combinations of three species as both target and neighbour species in order to address the following questions: (1) Do plant interactions between these three species depend on nutrient addition? (2) Does the relative yield per plant of three species depend on nutrient availability and competitive interactions? and (3) Which plant traits are related to competitive ability of *R*. *confertus*?

Specific hypotheses were as follows:

1. *R*. *confertus* would be the strongest competitor in both unfertilized and fertilized plots and would have higher seedling height and shoot biomass than non-invasive species especially at high nutrient availabilities
2. plants from nutrient fertilized plots would have higher specific leaf area (SLA), leaf macronutrient content than unfertilized ones and
3. under nutrient limitation all three species would have greater biomass allocation to roots.

## Results

### Soil solution

Increasing amounts of the fertilizer added to the soil, resulted in enhanced concentrations of N, P and K in the soil solutions (Table 1).

**Table 1:** Physicochemical properties (pH, organic matter content, and macronutrient content [N, P, K]) of the soil in the fertilized plots used in the field experiment measured before planting and at end of the experiment.

Chemical properties	Before planting	End of experiment
pH-H2O)	6.7	6.3
Organic matter (% dry weight)	5.5	5.6
Total nitrogen (N, %)	0.4	0.6
Phosphorus (P, mg/100 g)	6.2	7.5
Potassium (K, mg/100 g)	21.4	23.4
**Texture**		
Sand (1.000–0.050 mm) (%)	38.0	38.1
Silt (0.050–0.002 mm) (%)	54.4	54.4
Loam (<0.002 mm) (%)	7.6	7.5

### Seedling height

**Figure 1:**
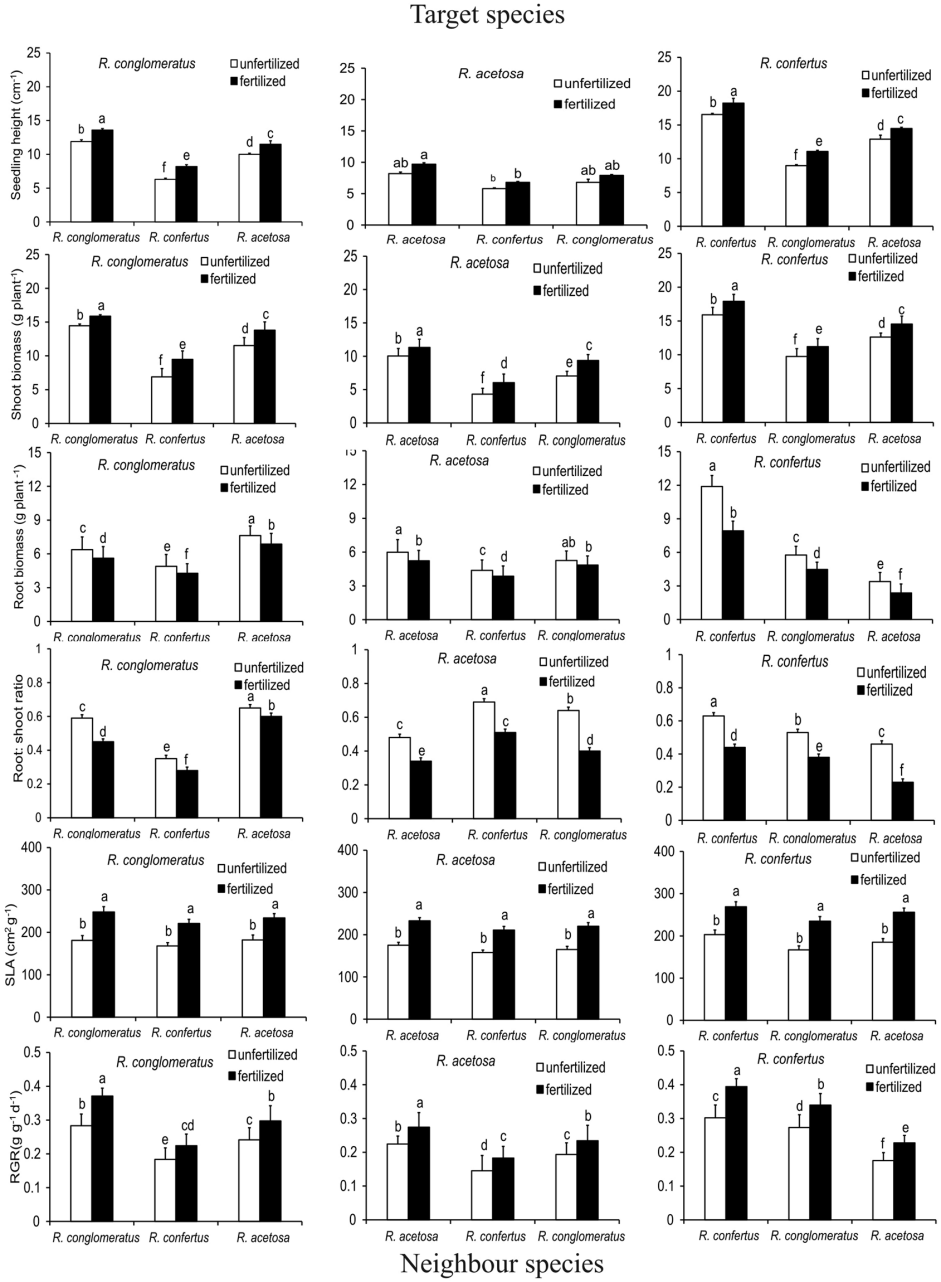
Effects of competition and nutrient on early growth of *Rumex conglomeratus*, *R*. *acetosa* and *R*. *confertus* in each of two nutrient treatments. (*n* = 5 per species and treatment). The results are means (±SD) for all combinations of target (arranged in columns) and neighbour species (in rows). Different letters denote significant differences between mean values (ANOVA, Tukey’s HSD post hoc test with Bonferroni correction at *P* < 0.01).

In unfertilized and fertilized plots, the height of both native species was significantly decreased by the invasive species (each *P* < 0.01). In addition the height of native species in the fertilized monoculture and in the mixture was significantly higher than without the fertilizer (Fig. 1). Analysis of variance showed that the seedling heights of *R*. *confertus*, *R*. *conglomeratus* and *R*. *acetosa* were influenced by competition (for all *P* < 0.001; Sypplementary Table S1). This analysis also revealed a significant interaction between competition and fertilization (for all *P* < 0.001; Table 2), i.e. fertilization mediated the effect of competition on the seedling height.

**Table 2:** Summary of two-way ANOVA results (*F*-values and significance levels) of seedlings traits (plant height, shoot and root biomass) or relative growth rate (RGR) in response to competition (presence−absence), fertilization (control vs fertilized) and their interaction in *Rumex conglomeratus* (a), *R*. *acetosa* (b) and *R*. *confertus* (c).

Source of variation	Seedling height			Shoot biomass			Root biomass			SLA			Root:shoot ratio			RGR		
	df	*F*	*P*	df	*F*	*P*	df	*F*	*P*	df	*F*	*P*	df	*F*	*P*	df	*F*	*P*
**(a)** ***R***. ***conglomeratus***																		
Competition (C)	1	18.6	<0.001	1	68.5	<0.001	1	9.7	<0.001	1	11.2	<0.01	1	10.2	<0.01	1	46.7	<0.01
Fertilization (Ft)	1	17.1	<0.001	1	57.3	<0.001	1	9.2	<0.001	1	7.8	<0.01	1	8.9	<0.01	1	62.3	<0.01
C × Ft	1	9.6	<0.001	1	35.5	<0.01	1	4.8	<0.001	1	12.5	<0.01	1	7.8	<0.01	1	36.3	<0.01
**(b)** ***R***. ***acetosa***																		
Competition (C)	1	17.9	<0.01	1	57.4	<0.001	1	8.8	<0.01		5.8	<0.01	1	6.6	<0.01	1	41.7	<0.001
Fertilization (Ft)	1	16.3	<0.001	1	49.2	<0.001	1	7.6	<0.001		5.6	<0.01	1	7.5	<0.01	1	53.5	<0.001
C × Ft	1	8.5	<0.001	1	45.8	<0.001	1	6.2	<0.001		9.2	<0.01	1	8.1	<0.01	1	43.8	<0.01
**(c)** ***R***. ***confertus***																		
Competition (C)	1	14.5	<0.001	1	18.5	<0.001	1	17.8	<0.001		8.5	<0.01	1	6.7	<0.01	1	47.1	<0.01
Fertilization (Ft)	1	15.1	<0.001	1	14.9	<0.001	1	16.4	<0.001		9.0	<0.01	1	8.3	<0.01	1	42.7	<0.001
C × Ft	1	5.3	<0.001	1	4.8	<0.001	1	5.7	<0.001		7.6	<0.01	1	7.5	<0.01	1	26.5	<0.01

Absence of competition refers to situations where seedlings were grown in monocultures; presence of competition refers to mixtures of seedlings. Data log transformed.

### Shoot biomass

Application of the fertilizer significantly increased (for all *P* < 0.01) the shoot biomass *R*. *conglomeratus* and *R*. *acetosa* in both monocultures and mixtures. For example, in monocultures the fertilized seedlings of *R*. *confertus*, *R*. *conglomeratus* and *R*. *acetosa* had respectively 18%, 17.6% and 22% higher shoot biomass compared to the unfertilized ones (for each *P* < 0.01). In both unfertilized or fertilized monocultures the shoot biomass of *R*. *confertus* significantly exceeded that of *R*. *conglomeratus* (*P* = 0.0023), which in turn significantly exceeded that of *R*. *acetosa* (*P* = 0.0121). In the mixture, *R*. *confertus* caused a marked reduction in the shoot biomass of *R*. *conglomeratus* or *R*. *acetosa* (Supplementary Table S1). For example, on the unfertilized plots, *R*. *confertus* reduced *R*. *acetosa* shoot biomass by 25%, a proportion significantly higher than that caused by *R*. *conglomeratus* (22%; Fig. 1). A two-way ANOVA showed that shoot biomass production depended on competition and fertilization (C × Ft: *F* = 4.8, *P* < 0.001; Table 2).

### Root biomass

In the unfertilized monoculture the root biomass of *R*. *confertus* significantly exceeded that of *R*. *conglomeratus* (*P* = 0.0013), which in turn significantly exceeded that of *R*. *acetosa* (*P* = 0.0191). In all three species root biomass decreased with increasing nutrient supply (*R*. *confertus*: *P* = 0.0019; *R*. *conglomeratus*: *P* = 0.0078; *R*. *acetosa*: *P* = 0.0035). When *R*. *confertus* competed with *R*. *acetosa* or *R*. *conglomeratus* root biomass of the later two was significantly reduced both with and without the fertilizer. However, the effect was more pronounced for *R*. *acetosa* than for *R*. *conglomeratus* (each *P* < 0.001; Fig. 1; Supplementary Table S1). A two-way ANOVA showed that fertilization had significant effect on root biomass of the invasive species (*P* < 0.001) and two native species (each *P* < 0.001; Table 2).

### Root: shoot ratio

All three *Rumex* species increased biomass partitioning to roots as nutrient supply decreased (Fig. 1). A two-way ANOVA showed that root: shoot ratio was influenced by competition (Supplementary Table S1), fertilization and the interaction between these factors (for all *P* < 0.01; Table 2).

### Specific leaf area

The increased nutrient supply increased SLA. Within mixtures competition with *R*. *confertus* significantly reduced SLA of two native species (Fig. 1). Analysis of variance (ANOVA) showed a significant effect of competition and fertilization on SLA of *R*. *confertus*, *R*. *conglomeratus* and *R*. *acetosa* (for all *P* < 0.01; Supplementary Table S1) with significant interaction between these factors (for all *P* < 0.01; Table 2).

### Relative growth rate (RGR)

RGR was influenced by competition and fertilization, with interaction between these factors (for all *P* < 0.01). Addition of the fertilizer increased RGR of all three species in the monoculture and the mixed treatments. However, in the monoculture *R*. *confertus* showed the greatest RGR among all species under both nutrient treatments. Compared with the monoculture, *R*. *confertus* reduced RGR of *R*. *conglomertaus* and *R*. *acetosa* under both unfertilized and fertilized conditions (Fig. 1; Supplementary Table S1). To summarize, RGR of all species increased with nutrient availability (ANOVA, for all post hoc *P* < 0.01; Table 2).

### Leaf macronutrient (N, P, K) and nitrate (NO_3_^−^-N) contents

**Figure 2:**
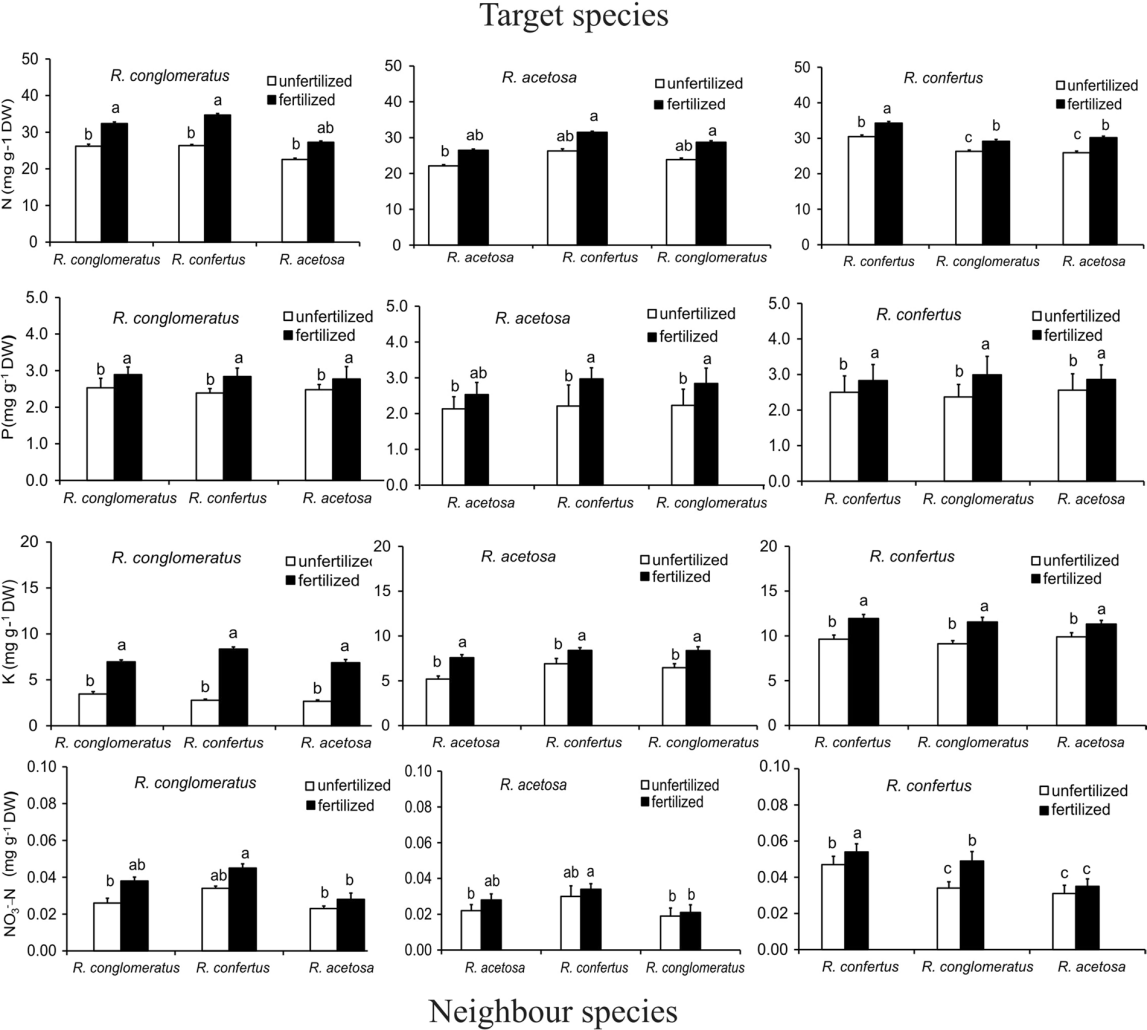
Effects of competition and nutrient fertilizer on N, P, K and NO_3_^−^-N contents of leaves of *Rumex conglomeratus*, *R*. *acetosa* and *R*. *confertus* seedling in each of two nutrient treatments. (*n* = 5 per species and treatment). The results are means (±SD) for all combinations of target (arranged in columns) and neighbour species (in rows). Different letters denote significant differences between mean values (ANOVA, Tukey’s HSD post hoc test with Bonferroni correction at *P* < 0.01). In leaves the nutrient contents per plant estimated as the product of nutrient concentration, expressed as percentage of dry matter obtained from three leaves per plant.

Analysis of variance (ANOVA) of N, P, K or NO_3_^−^-N contents indicated significant effects of fertilization. In addition, ANOVA showed that in all mixtures, competition had no effect on leaf nutrient content indicating that N, P, K or NO_3_^−^-N content in *R*. *conglomeratus* or *R*. *acetosa* when grown with *R*. *confertus* were not significantly different from those in monocultures (Supplementary Table S2. However, in the fertilized plots the leaf macronutrient or NO_3_^−^-N contents of *R*. *confertus* plants increased to a greater extent than those of *R*. *acetosa* and *R*. *conglomerates* plants. For example, in monoculture, leaf NO_3_^−^-N content of *R*. *confertus* was 46% higher in the fertilized plots as compared to the unfertilized ones, whereas in *R*. *acetosa* the increase was only 27% (Fig. 2; Table 3).

**Table 3:** *F*-values and significance levels from two-way analyses of variance (ANOVA) concerning macronutrient (N, P, K) or nitrate (NO_3_^−^-N) concentrations in leaves of *Rumex conglomeratus* (a), *R*. *acetosa* (b) and *R*. *confertus* (c).

Source of variation	N			P			K			NO_3_^−^-N		
	df	*F*	*P*	df	*F*	*P*	df	*F*	*P*	df	*F*	*P*
**(a)** ***R***. ***conglomeratus***												
Competition (C)	1	2.3	>0.05	1	11.3	>0.05	1	8.3	>0.05	1	3.6	>0.05
Fertilization (Ft)	1	11.1	<0.01	1	10.5	<0.01	1	16.0	<0.01	1	11.5	<0.01
C × Ft	1	5.6	<0.05	1	6.0	<0.05	1	5.6	<0.05	1	10.3	<0.01
**(b)** ***R***. ***acetosa***												
Competition (C)	1	3.4	>0.05	1	16.5	>0.05	1	6.6	>0.05	1	5.3	>0.05
Fertilization (Ft)	1	15.4	<0.01	1	14.7	<0.01	1	12.8	<0.01	1	13.6	<0.01
C × F	1	11.6	<0.05	1	8.5	<0.01	1	8.8	<0.05	1	9.7	<0.01
**(c)** ***R***. ***confertus***												
Competition (C)	1	4.4	>0.05	1	28.5	>0.05	1	3.1	>0.05	1	2.2	>0.05
Fertilization (Ft)	1	16.3	<0.01	1	17.2	<0.01	1	25.4	<0.01	1	9.3	<0.01
C × Ft	1	7.9	<0.01	1	10.5	<0.01	1	9.8	<0.05	1	7.5	<0.01

Main effects are F (fertilization treatment) and C (competition). Interaction C × F between fertilization treatments and competition are also given. Data log transformed.

### Relative yields

Based on the observed changes in the total biomass of the harvested plants under both nutrient treatments the relative yields of *R*. *acetosa* in relation to *R*. *confertus* (RY_acetosa+confertus_) and of *R*. *conglomeratus* to *R*. *confertus* (RY_conglomeratus+confertus_) were significantly (in both *P* < 0.01) smaller than 1. So *R*. *confertus* outperformed both *R*. *acetosa* and *R*. *conglomeratus* in competition. Moreover, under unfertilized and fertilized conditions the relative yield of *R*. *conglomeratus* in relation to *R*. *acetosa* (RY_conglomeratus+acetosa_) was significantly higher (*P* < 0.01) than 1 which implies that *R*. *conglomeratus* outperformed *R*. *acetosa* in competition (Table 4).

**Table 4:** Relative yields (RY_ab_) of three *Rumex* species in mixture based on total biomass of single plants in relation to nutrient fertilizer (n = 5).

Fertility treatment	Unfertilzed	Fertilized
RY_confertus+acetosa_	0.54	0.69
RY_confertus+conglomeratus_	0.58	0.75
RY_acetosa+confertus_	0.61	0.89
RY_conglomeratus+confertus_	0.69	0.92
RY_conglomeratus+acetosa_	1.21	1.46
RY_acetosa+conglomeratus_	0.58	0.83

## Discussion

The competitive effects of invasive *R*. *confertus* on two native species, *R*. *conglomeratus* and *R*. *acetosa* were examined at different nutrient levels. Competitive ability can be measured in various ways^45^. In the present study, it was evaluated by calculating the relative yield (RY) per plant for each species. As predicted (hypothesis 1) the invasive *R*. *confertus* outperformed the native *R*. *conglomeratus* and *R*. *acetosa* in competition at both low (control treatment) and high nutrient availability. Plant size (plant height) was shown to be highly correlated with competitive ability^46^. For example, Gaudet and Keddy^47^ comparing the short-term effects of 44 wetland species on the biomass of a phytometer (a plant on which the competitive effect of a test plant is measured), showed that the competitive ability of the test plants relative to the phytometer was highly correlated with their above-ground biomass and associated traits, such as plant height. Although *R*. *confertus* was not the tallest at the beginning of the experiment, it overtopped both native species rather early. Thus *R. confertus* is a strong competitor against its shorter native species, especially *R. acetosa*, which was suppressed by *R. confertus* in terms of all traits measured (hypothesis 1). In fertile habitats where canopies are well developed, taller plants have an advantage of gaining light in competition with shorter neighbours^48^.

In all experiments, RY of *R. confertus* was <1, indicating that for this species the effects of interspecific competition were greater than those of intraspecific competition^38^. The relative yields of *R*. *acetosa* in relation to *R*. *confertus* (RY_acetosa+confertus_) or of *R*. *conglomeratus* in relation to *R*. *confertus* (RY_conglomeratus+confertus_) on the unfertilized plots were smaller than on the fertilized ones. So, interspecific competition was lower on fertilized plots because RY was closer to 1. According to Grime^2^ competition is more intensive at high nutrient levels, which might explain low species diversity in nutrient-rich grassland ecosystems. But, on the other hand Tilman^49^ stated that the intensity of competition was either independent of or decreases with soil fertility.

In this study nutrient supply increased growth of all three *Rumex* species but the effect was most pronounced for the invasive *R*. *confertus*. A number of other examples were reported in which the competitive ability of a component species was altered by nutrient availability, for example between invasive grass *Phragmites australis* and a native competitor *Spartina* pectinata^36^, between *Daucus carota* and *Chenopodium album*^50^, between invasive *Hydrocotyle vulgaris* and terrestrial species^51^ and among nutrient-limited heachland plants^38^.

The shoot biomass of all three species was strongly influenced by competition especially in the high-nutrient treatment. The shoot biomass of native *R*. *acetosa* was reduced in competition with both congeners which shows that *R*. *acetosa* is the weakest competitor of the three species. The shoot biomass of *R*. *conglomeratus* decreased in two nutrient treatments in competition with *R*. *confertus* and increased in competition with *R*. *acetosa*. This indicates an intermediate position of native *R*. *conglomeratus* in the competitive hierarchy within the members of genus *Rumex*. The shoot biomass of *R*. *confertus* was not significantly affected by the congeners, which also indicates this species competitive superiority.

Three species investigated in this study exhibited significant differences in growth attributes. As predicted (hypothesis 2) SLA of the studied species increased with N, P, K supply. In addition invasive *R*. *confertus* had higher SLA than its non-invasive congeners, *R*. *conglomeratus* and *R*. *acetosa* under the same conditions. These results are consistent with those of other studies that reported that high SLA might be associated with invasive species^52–54^. Leaves with higher SLA have higher N concentrations^55^, leading to higher respiration^55^, carbon assimilation and RGR^56^. Therefore, high SLA values are characteristic of faster-growing species^57^. Differences in SLA between species might be caused by differences in leaf thickness or leaf tissue composition. For example leaf tissue density will be greater in the leaves with higher concentration of phenolic compounds (lignin and tannins), or secondary metabolites, resulting in lower SLA^57^. Experimental studies showed that RGR may be a better indicator of invasiveness than SLA. For example, SLA was more closely correlated with invasiveness than RGR for *Acacia* and *Acer*, while RGR was more tightly associated with invasiveness for the Rosaceae^53^. Invasive species achieved higher RGR than natives primarily by having a higher net rate of dry matter production and/or lower rates of respiration (high NAR, net assimilation ratio) by allocating more biomass to leaves (high LMR, leaf mass ratio), or producing thinner or less dense leaves resulting in more leaf area per unit leaf biomass (high SLA)^58^. As Grime and Hunt^59^ showed RGRs of 130 herbaceous species and tree seedlings in the local flora of Sheffield, England, ranged from 0.031 to 0.314 g g^−1^ d^−1^ (mean = 0.152). In the present study, mean RGR of *R*. *confertus* seedlings in the high-nutrient treatment was 0.35 g g^−1^ d^−1^, thus, it was above of the range of RGRs of the 130 species studied by Grime and Hunt^59^. High seedling RGR under non-limiting resources was found to be the most important trait of six species of dayflowers (Commelinaceae)^60^, four species of *Senecio* (Asteraceae)^61^ and of pine species^53^. A few other comparisons of RGRs of invasive and non-invasive congeners also generally showed that invasive plants had higher RGR than the native ones^29,53,60^. In contrast, Bellingham *et al*.^62^ did not find any correlation between invasiveness and RGR for seedlings of 33 woody species of gymnosperms and angiosperms. In this study, at both low and high level of fertility I expected *R*. *confertus* to be the superior competitor, with *R*. *conglomeratus* second and *R*. *acetosa* third. The results were as expected in all the species combinations. This suggests that *R*. *confertus* has a higher output per unit input and such a species is able to dominate an area quickly. Higher RGR gives *R*. *confertus* competitive advantage allowing it to pre-empt resources at an early stage of the growing season.

Phenotypic plasticity can be broadly defined as the ability of plants to alter their morphology and/or physiology in response to varying environmental conditions it may increase the competitive ability of a plant over a range of different resource availabilities^63,64^. The two common native species, *R*. *acetosa* and *R*. *conglomeratus*, as well as the invasive species *R*. *confertus* allocated relatively more biomass to the roots at low nutrient supply (Fig. 1), possibly increasing their competitive ability for belowground resources. However, both in monocultures or in mixtures the percentage decrease in biomass allocation to the roots in *R*. *confertus* exceeded those in both native species (Fig. 1) thus pointing to its higher phenotypic plasticity regarding the partitioning of biomass between shoots and roots.

Similar pattern was found by Aerts *et al*.^38^ in evergreen shrubs, *Erica tetralix* and *Calluna vulgaris* and perennial grass, *Molinia caerulea*. High plasticity of *R*. *confertus* may have significant effects on seedling establishment and competitive ability, and hence on species distribution^65^.

*Rumex confertus* showed significantly higher values of N, P, K or NO_3_^−^-N content, than the other Species. Nazaryuk *et al*.^66^ showed that the nitrate accumulation in plants depended on three major groups of factors: the amount and kind of a fertilizer applied, treatment with physiologically active substances, and natural and anthropogenic changes in the soil environment. All these factors may be arranged in the following order: fertilizers > physiologically active substances > soil. Nitrate accumulation in plants increased with nitrogen supply, whereas limiting the nitrogen availability reduced nitrate content significantly (see Umar and Iqbar^67^ for review). Both net absorption and assimilation rates during the growing season are genetically determined, explaining a large variability of plant nitrate content among plant species, and even among cultivars of the same species (see Cárdenas-Navarro *et al*.^68^ and references therein). This study showed that the nutrient content levels in the leaves were within the range of the reference values known for *R*. *crispus*^69^ and for *R*. *alpinus*^70^. Changes in the biomass of shoots and roots caused by species competition, did not translate into changes of the amounts of N, P, K or NO_3_^−^-N stored in the leaves. This is consistent with Zaller^71^ results, who tested the competitive ability of *R*. *obtusifolius* against grassland species.

Differences between species in root biomass are associated with environmental conditions. Boot and Mensink^72^ found that species from fertile sites had higher root: shoot ratios at both low and high N supply than did species from infertile sites. Others authors reported that fast-growing species from fertile sites generally had higher capacity to adjust mass allocation (see Hill *et al*.^73^ for the list and references). There are reports concerning many higher plant species showing decreased root: shoot ratio correlated with increased growth due to higher nutrient supply^74^. These results are consistent with the results presented here because the tested species were generally able to modify their root systems in response to nutrient. The hypothesis 3 in the studied species, the relative amount of biomass allocated to the roots was lower in the fertilized variant was confirmed. These findings are similar to those reported by McConnaughay and Coleman^74^ for the weed species *Abutilon theophrasti*, *Chenopodium album* and *Polygonum pensylvanicum*.

In central Poland, damp meadows on which *R*. *confertus* grows are often located below the fields and mineral fertilizers from them flow with water to these neighboring meadows. Thus its success may result from positive response to high nutrient availability. This is consistent with the pattern found in *P*. *australis* by Rickey and Anderson^36^.

## Conclusion

The high invasiveness of *R*. *confertus* seems to result from its competitive dominance over the other congeners across a very wide range of nutrient availability. The high competitive ability of *R*. *confertus* with respect to both *R*. *acetosa* and *R*. *conglomeratus* was caused by (a) higher SLA, (b) great plant height, and (c) higher potential growth rate. This combination of plant traits reduces light absorption or photosynthate production of *R*. *acetosa* and *R*. *conglomeratus* thereby limiting their growth.

With strengthened competitive ability of *R*. *confertus*, weakened competitive ability in the other species (*R*. *acetosa* and *R*. *conglomeratus*) and consequent changes in competitive hierarchy, I expect possible changes in species dominance and structure of meadow plant community in central Europe in the future.

## Materials and Methods

### Study species

The studied *Rumex* (Polygonaceae) species are biennials with similar life-histories and reproductive characteristics, they coexist in some habitats but with different origin and invasion status in Poland. *Rumex acetosa*, *R*. *conglomeratu*s and *R*. *confertus* were chosen to test interspecific competition because these species are the most common neighbors in moist to wet meadows and pastures in Poland. All three studied *Rumex* species reproduce clonally (ramets produced from root and rhizome buds) and sexually from seeds.

According to their ecological indicator values^75^, *R*. *acetosa* (Ellenberg’s N-value = 6) and *R*. *conglomeratus* (Ellenberg’s N-value = 8) show similar requirements for soil nutrients. Generally, both species are indicators of high soil nitrogen concentrations^75^.

*Rumex confertus* is native to temperate Eastern Europe and Asia where it thrives on meadow-steppes and glades in forest-steppe. Its height varies from 60 to 120 cm. In Poland it occurs predominantly along rivers and invades seminatural vegetation (e.g. meadows, wet ditches, riparian-scrub), but has been recently colonizing disturbed habitats (such as roadsides, railway tracks and embankments), forest clearings and margins^76,77^. The aggressiveness of *R*. *confertus* results from its ability to establish quickly from seed, to flower in its first year and to its fast growth and high seed production some of which can remain viable for very long periods in the soil^77^.

*Rumex conglomeratus* grows in wet meadows, stream and river banks, ditches, field margins and gateways, often in places flooded or waterlogged in winter^78^. This species is characterized by a height similar to that of *R*. *con*fertus and a broad ecological amplitude. This Eurosiberian Southern-temperate element became widely naturalised so that distribution is now Circumpolar Southern-temperate^78^.

The third species, *R*. *acetosa* occurs in a wide range of habitats, but particularly at waste ground, road sides, disturbed areas, grasslands (mainly pastures), and arable land^78^. Its height varies from 30 to 80 cm. *Rumex acetosa* occurs in meadows throughout Europe but rarely in the south as well as in parts of Central Asia. It occurs as an introduced species in parts of North America^79,80^.

### Plant material

Freshly matured seeds of *R*. *acetosa*, *R*. *conglomeratus* and *R*. *confertus* were collected in a wet pasture on 2 September 2016 in the vicinity of Uniejów (51°96′N, 18°79′E), 50 km west of Lodz, central Poland. For each species, I collected a mixed sample of seeds from at least 20 individuals randomly chosen from the whole site. The seeds were stored in paper bags at 4 °C until use.

### Experimental design and growth conditions

An experiment was conducted in the experimental garden on a private property in the vicinity of Uniejów, 60 km west of Lodz, where the mean annual temperature was 8.8 °C and mean annual precipitation (rain and snow) was 587.2 mm (meteorological data, Lodz station). The seeds were stratified in a refrigerator at 4 °C for a 16 weeks and then germinated on moist filter paper in a Petri dish in growth cabinet (16 h light, 24 °C, 20 μmol m^−2^ s^−1^ PPFD; 8 h dark, 10 °C). After three weeks, uniform seedlings with two leaves were selected and transplanted into plastic pots 25 cm in diameter, 22 cm deep (1962 cm^2^) filled with a mixture (1: 4, v/v) of sterilized soil and sterilized sand and cultured in a growth room (23 °C; 14 light/10 h dark; 80 μmol m^−2^ s^−1^ PPFD, Philips TL 94,) for three months.

Next the same size seedlings of individual species were transferred to field plots, 22 days after germination ([T_1_], 17 April 2017). Seedling biomass (±SD) at the start of the experiment (T_1_) was equal for *R*. *confertus* (0.41 ± 0.05 g) and *R*. *conglomeratus* (0.39 ± 0.04 g) while *R*. *acetosa* seedlings had smaller mass (0.28 ± 0.02 g). After planting, the seedlings were grown in the open air during May−August a period of moderate temperature (Table 5).

**Table 5:** Mean air temperature at the field site during *Rumex* seedling growing period for 153 days from 17 April to 17 August 2017.

		April	May	June	July	August
Temperature °C	Daily maximum	13.8	18.7	22.0	24.0	23.3
	Daily minimum	3.9	7.9	11.3	13.4	12.8
	Daily mean	8.8	13.3	16.6	18.7	18.0

The plants were planted in monocultures and mixtures using a replacement design: each species in monoculture and also in a 1:1 mixture of each of two species. All treatments were carried out with four replicate plots in a randomized block design. A total number of 48 plots (2 nutrient treatments × 6 species-competition combinations × 4 replicates) were used for the experiment.

**Figure 3:**
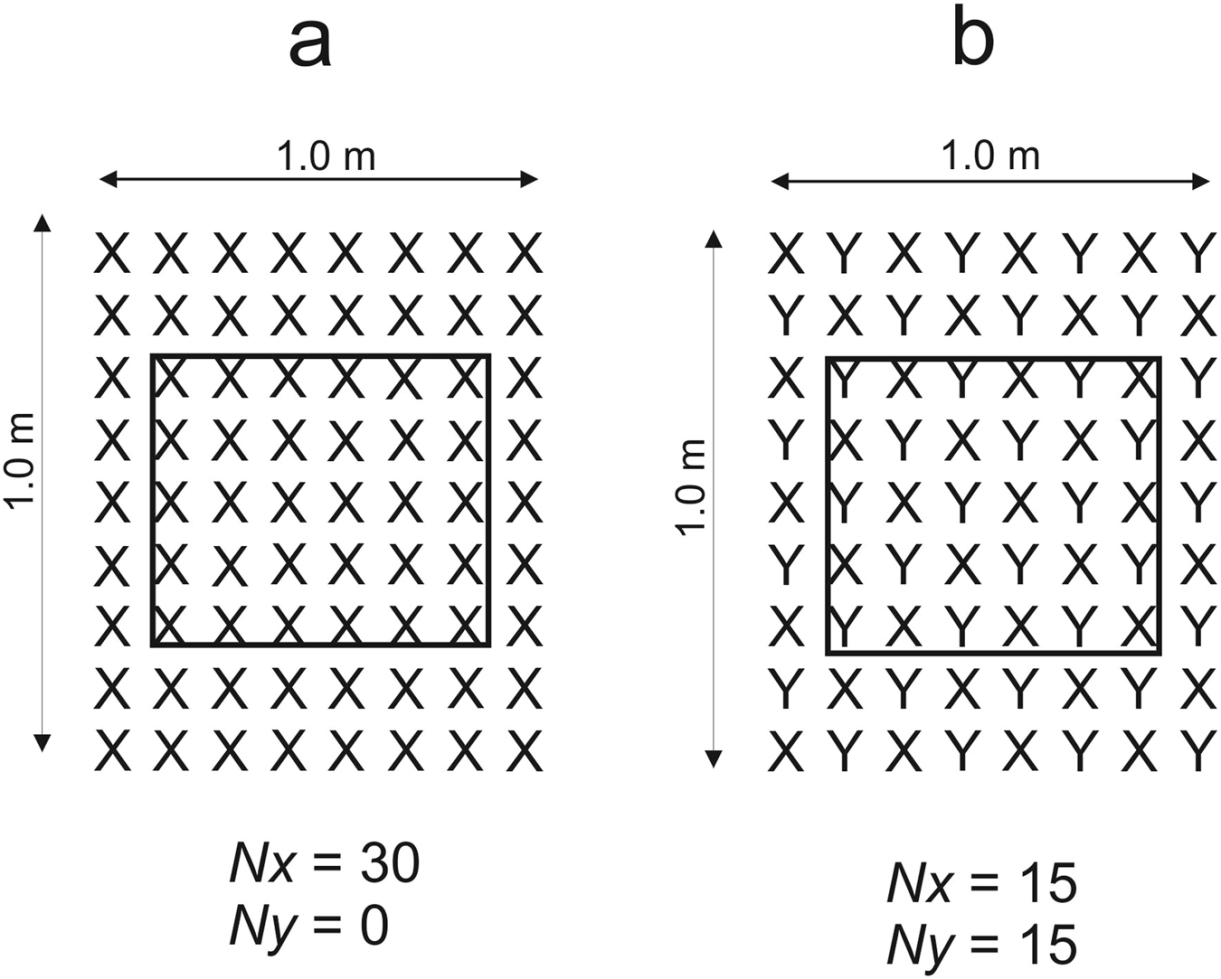
Diagram of the arrangement of plants in the plots for the total density of 72 plants m^2^. Each symbol represents a single plant, separated from its neighbors by 10 cm. (**a**) species *x* in monoculture, (**b**) species *x* in mixture with species *y*. *N*_*x*_ = number of plants of species *x*; *N*_*y*_ = number of plants of species *y*.

To avoid border effects, only the inner 30 individuals of each plot (6 rows of 5 plants in) in both monocultures or in mixtures were analyzed (Fig. 3).

Two nutrient treatments for each species-competition combination were imposed after transplanting the seedlings into plots: unfertilized (control) and fertilized. The commercial (Inco VERITAS S.A., Poland) fertilizer “Azofoska” (40 g m^−2^ yr^−1^) containing (%): 5.5 NO_3_, 8.1 NH_4_, 6.4 P_2_O_5_, 19.1 K_2_O, 4.5 MgO, 0.27 Fe, 0.045 Mn, 0.18 Cu, 0.045 B, 0.082 Mo, 0.045 Zn was used. The fertilizer was evenly spread by hand and was supplied twice during the growing season, on 25 May and 25 June (20 g each time).

The plots (1 × 1 m) were separated by equally sized (0.5 m wide) buffer zones. There were 8 rows of 9 seedlings in both monocultures and mixture plots. However, plant density of each species in the mixture was half of the density of that species in monoculture. In the plots containing mixtures of seedlings, 36 seedlings of each of the two species were placed alternately in rows at the spacing of 10 cm. Thus each seedling (except at the margin) was surrounded by four seedlings of the other species. To avoid border effects, only the inner 30 individuals of each plot (6 rows of 5 plants: both in monocultures and mixtures) were analyzed (Fig. 3). Watering to field capacity, using tap-water, was carried out every three days.

### Soil physical and chemical analysis

In each plot one soil core (3 cm diameter × 5 cm deep) was collected at random and put in a plastic bag. Soil cores were mixed by hand up into a single bulk sample for each plot. Then the soil samples were air-dried (20–25 °C for 3 days) to a constant weight and sieved through a 2-mm mesh screen. The pH was determined in water (ratio 1:1, soil: water). The soil samples (100 g dry soil) were analysed for selected macronutrients: total nitrogen (N), phosphorus (P) and potassium (K). Phosphorus was measured by colorimetric molybdenum blue method in 0.5 M sodium bicarbonate (NaHCO_3_) extract of soil. Potassium was extracted from soil with calcium lactate (C_6_H_10_CaO_6_) and determined by atomic absorption spectrometer (AAS) method. Total N was determined by the micro-Kjeldahl procedures by the wet oxidation of organic matter using sulfuric acid (H_2_SO_4_) and a digestion catalyst. The amount of soil organic matter was estimated as loss on ignition (550 °C, 3 h) and calculated as % dry weight (Table 2). The analyses were performed at the Chemical-Agricultural Station in Lodz.

### Harvest and biomass sampling

At the end of the experiment ([T_2_]; 17 August 2017, 16 weeks following transplantation) height (cm) of the main shoot of each seedling was recorded before they were harvested. In general, ten whole plants of each species-competition combination were randomly collected within both unfertilized and fertilized plots. A total number of 240 seedlings per species (2 nutrient treatments × 3 species combination × 4 replicates × 10 seedlings) were collected for the experiment. To measure shoot biomass, each species shoot (leaves + stem) was cut off at the soil surface. Roots and shoot were carefully washed in the lab. Leaf area was measured on the same day with a CI-202 portable laser leaf area meter (CID Bio-Science, USA). After determination of the area, the leaves were dried for at least 48 h at 70 °C and dry mass was determined. Specific leaf area (SLA; cm^2^ g^−1^) was calculated as area per unit mass. Shoots and roots of a single plant were dried to a constant weight for at least 72 h at 70 °C in an oven, and weighed (g). The relative growth rate (RGR) was estimated using the following formula:

1 $$RGR=(ln{W}_{t2}-ln{W}_{t1})/({T}_{2}-{T}_{1})$$

where, *W*_*t2*_ *W*_*t1*_ *are plant* dry weight (DW in mg) of seedlings at time T_1_ and time T_2_, respectively^81^.

### Relative yield

The effect of competitive response (neighbours) was quantified using relative yield per plant for each species^82^. The relative yield (RY_ab_) was calculated as:

2 $${{\rm{RY}}}_{{\rm{ab}}}={{\rm{Y}}}_{{\rm{ab}}}/{{\rm{Y}}}_{{\rm{a}}}$$

where, Y_ab_ is the dry biomass yield of species *a* in mixture with species *b* and Y_a_ is the dry biomass yield of species *a* in monoculture. If RY = 1, there is absolutely no competition. If RY_ab_ > 1 species *a* outperformed species *b* in competition, when RY_ab_ < 1 the reverse is true^38^.

### Foliar macronutrient and nitrate analyses

Samples of three (youngest fully expanded) leaves were collected from each of 10 different plants separately for each competition treatment. Pooling of three leaves on a plant was necessary to obtain enough tissue for the macronutrient (N, P and K) and nitrate (NO_3_^−^-N) analyses. Afterwards, the leaf samples were dried (105 °C for 5 h) and ground with a plant-sample mill and siewed with a 2-mm mesh screen before the sub-samples were weighed with a precision balance. Prior to chemical analyses, the leaf samples of each harvest were combined into four independent sub-samples (100 mg of each). Two thirds being used for macronutrient determination and one third − for nitrate determination. The leaf material was mineralized in a boiling mixture of 10 ml of HNO_3_ and 5 ml of H_2_SO_4_. Phosphorus was determined colorimetrically (see soil properties), potassium by atomic absorption spectrometry (as above), and the total nitrogen content by the micro-Kjeldahl method (as above). Nitrate concentration in plant tissue was determined with a Corning NO_3_^−^ ion selective electrode (Orion Analyzer Application Bull. No. 7, Determination of nitrate in plant tissue). Leaf macronutrients and tissue NO_3_^−^-N contents were as calculated as the total amount of macronutrients and nitrate per unit of dry leaf mass (mg/g dry wt^−1^).

### Data analysis

Normality was verified with the Shapiro-Wilk test. All data were log transformed prior to the analysis if necessary to meet the assumption of normality. Student’s t-test was performed to test for statistical differences in relative yields (RY_ab_) between unfertilized and fertilized plots. For unfertilized and fertilized plots, differences between species in seedling height, shoot biomass, root biomass, SLA and RGR were analysed by one-way ANOVA with post-hoc Tukey’s range tests. The effect of competition (monoculture vs mixture plots), fertilization (control vs fertilized) and their interaction on seedlings traits (plant height, shoot and root biomass) or relative growth rate (RGR) for each species was analyzed using a two-way nested ANOVA. Two-way ANOVA was also performed to test the effect of competition (monoculture vs mixture plots), fertilizatiom (control vs fertilized) and their interaction on concerning macronutrients (N, P, K) or nitrate (NO_3_^−^-N) concentrations in leaves of three species. Statistical analyses were performed using the Statistica 13.0 package^83^.

## Supplementary information

Supplementary Tables

## Data Availability

All data generated or analysed during this study are included in this published article (and its Supplementary Information File).
