# Supplementary material for: Growth and competitive interaction between seedlings of an invasive Rumex confertus and of co-occurring two native Rumex species in relation to nutrient availability
Source: Sci Rep. 2019 Mar 1;9:3298. doi: 10.1038/s41598-019-39947-z (PMC6397286; doi:10.1038/s41598-019-39947-z)
Supplement: Supplementary file 1 — Supplementary Tables [file 41598_2019_39947_MOESM1_ESM.docx]

**Supplementary Tables**

Article in Scientific Reports

**Growth and competitive interaction between seedlings of an invasive Rumex confertus and of co-occurring two native Rumex species in relation to nutrient availability**

Jeremi Kołodziejek

Department of Geobotany and Plant Ecology, University of Lodz, 12/16 Banacha St., 90-237, Lodz, Poland.

Correspondence should be addressed to J.K. (email: jeremi.kolodziejek@biol.uni.lodz.pl)

**Table S1.** Summary of the one-way ANOVA results (*F*-values and significance levels) of seedlings traits (plant height, shoot and root biomass) or relative growth rate (RGR) of *Rumex conglomeratus*, *R. acetosa* and *R. confertus*.

| **Source of variation** | **Seedling height** | | | **Shoot biomass** | | | **Root biomass** | | | **SLA** | | | Root:shoot ratio | | | **RGR** | | |
| --- | --- | --- | --- | --- | --- | --- | --- | --- | --- | --- | --- | --- | --- | --- | --- | --- | --- | --- |
|  | **df** | ***F*** | ***P*** | **df** | ***F*** | ***P*** | **df** | ***F*** | ***P*** | **df** | ***F*** | ***P*** | **df** | ***F*** | ***P*** | **df** | ***F*** | ***P*** |
| *R. conglomeratus* | 5 | 11.5 | < 0.001 | 5 | 12.1 | < 0.001 | 5 | 16.6 | < 0.001 | 5 | 28.4 | < 0.01 | 5 | 29.2 | < 0.01 | 5 | 38.5 | < 0.01 |
| *R. acetosa* | 5 | 13.6 | < 0.001 | 1 | 9.3 | < 0.001 | 5 | 16.6 | < 0.001 | 5 | 19.6 | < 0.01 | 5 | 27.4 | < 0.01 | 5 | 48.2 | < 0.01 |
| *R. confertus* | 5 | 10.3 | < 0.001 | 1 | 7.1 | < 0.001 | 5 | 21.6 | < 0.001 | 5 | 25.1 | < 0.01 | 5 | 26.3 | < 0.01 | 5 | 32.9 | < 0.01 |

**Table S2.** *F*-values and significance levels from the one-way analyses of variance (ANOVA) concerning macronutrient (N, P, K) or nitrate (NO_3_^−^-N) concentrations in leaves of *Rumex conglomeratus*, *R. acetosa* and *R. confertus*.

| **Source of variation** | **N** | | | **P** | | | **K** | | | **NO_3_^−^-N** | | |
| --- | --- | --- | --- | --- | --- | --- | --- | --- | --- | --- | --- | --- |
|  | **df** | ***F*** | ***P*** | **df** | ***F*** | ***P*** | **df** | ***F*** | ***P*** | **df** | ***F*** | ***P*** |
| *R. conglomeratus* | 5 | 31.3 | > 0.05 | 5 | 16.9 | > 0.05 | 5 | 23.7 | > 0.05 | 5 | 16.3 | > 0.05 |
| *R. acetosa* | 5 | 23.5 | > 0.05 | 5 | 25.7 | > 0.05 | 5 | 25.2 | > 0.05 | 5 | 17.2 | > 0.05 |
| *R. confertus* | 5 | 30.7 | > 0.05 | 5 | 28.5 | > 0.05 | 5 | 18.6 | > 0.05 | 5 | 15.3 | > 0.05 |
